# Functional Assessment of Genetically Modified Infrapatellar Fat Pad Mesenchymal Stem/Stromal Cell-Derived Extracellular Vesicles (EVs): Potential Implications for Inflammation/Pain Reversal in Osteoarthritis

**DOI:** 10.3390/cells14241952

**Published:** 2025-12-09

**Authors:** Kevin Liebmann, Mario Castillo, Stanislava Jergova, Behnaz Rahimi, Lee D. Kaplan, Thomas M. Best, Jacqueline Sagen, Dimitrios Kouroupis

**Affiliations:** 1 Department of Orthopedics, UHealth Sports Medicine Institute, Miller School of Medicine, University of Miami, Miami, FL 33146, USA; kxl403@med.miami.edu (K.L.); mxc2845@med.miami.edu (M.C.); kaplan@med.miami.edu (L.D.K.); txb440@med.miami.edu (T.M.B.); 2 Diabetes Research Institute & Cell Transplant Center, Miller School of Medicine, University of Miami, Miami, FL 33136, USA; 3 Miami Project to Cure Paralysis, Miller School of Medicine, University of Miami, Miami, FL 33136, USA; sjergova@med.miami.edu (S.J.); bxr658@med.miami.edu (B.R.); jsagen@med.miami.edu (J.S.)

**Keywords:** osteoarthritis, extracellular vesicles, CGRP, substance P, neuroinflammation

## Abstract

Osteoarthritis (OA) is a debilitating joint disease affecting over 500 million people globally, characterized by cartilage degradation, chronic pain, and failed tissue repair. Neurogenic inflammation, driven by neuropeptides including Substance P (SP) and calcitonin gene-related peptide (CGRP), plays a key role in the pathogenesis of OA. This study explores the therapeutic potential of extracellular vesicles (EVs) derived from infrapatellar fat pad mesenchymal stem/stromal cells (IFP-MSCs) transduced with CGRP antagonist CGRP_8-37_ (aCGRP IFP-MSC EVs). These EVs are enriched in anti-inflammatory miRNAs and proteins, and they express neprilysin (CD10), enabling SP degradation. Herein, several LncRNAs were identified, which have been known to interact with miRNAs that affect the knee joint homeostasis. Specifically, 11 LncRNAs (ZFAS1, EMX2OS, HOTAIRM1, RPS6KA2-AS1, DANCR, LINC-ROR, GACAT1, GNAS-AS1, HAR1A, OIP5-AS1, TERC) interact with miRNAs that promote cell proliferation, prevent apoptosis, and preserve homeostasis. In vitro, aCGRP IFP-MSC EVs downregulated pro-inflammatory markers (TNF, TLR4, MAPK8) in dorsal root ganglia and promoted chondrocyte gene expression consistent with anabolism and matrix remodeling. In vivo, intra-articular EV delivery attenuated pain behaviors, preserved the cartilage structure, restored PRG4+ stem/progenitor cell localization, and trended toward reduced SP levels. Histological analysis confirmed improved collagen organization and reduced matrix degradation. These findings suggest that aCGRP IFP-MSC EVs exert multimodal effects on neuroinflammation, cartilage regeneration, and joint homeostasis. This cell-free, gene-enhanced EV therapy offers a promising disease-modifying strategy for the treatment of OA, with the potential to address both structural changes and chronic pain associated with this disease.

## 1. Introduction

Knee osteoarthritis (OA) affects over 500 million people worldwide, contributing to disability and economic burden [1]. According to the WHO, about 73% of people living with OA are older than 55 years, and 60% are female. The pathogenesis of OA includes nociceptive signaling, neurogenic inflammation, and immune dysregulation [2]. The synovium and infrapatellar fat pad (IFP) become sites of immune infiltration, releasing pro-inflammatory and cartilage-degrading molecules, along with Substance P (SP) and calcitonin gene-related peptide (CGRP)—two neuropeptides central to pain sensitization and joint degeneration [3,4].

SP plays a critical role in nociception [5] and promotes local inflammation by increasing vascular permeability and driving macrophage polarization towards the M1 pro-inflammatory phenotype, which can affect synovial inflammation and cartilage degradation—key processes in the progression of OA [4,6,7,8]. Similarly, CGRP contributes to both central and peripheral pain mechanisms, with increased CGRP immunoreactive nerve fiber density observed in OA synovial tissue [9,10,11,12]. In OA, CGRP levels rise in response to joint injury, amplifying pain signaling and contributing to aberrant bone remodeling by inhibiting osteoclastogenesis, which may accelerate sclerosis and disease progression [13].

CGRP_8-37_, a CGRP antagonist (aCGRP), has exhibited analgesic effects across multiple pain models [14,15,16,17,18,19,20,21,22]. Gene therapy approaches using viral vectors, including adeno-associated virus (AAV), offer a promising strategy for long-term CGRP_8-37_ expression, with an excellent safety profile in over 130 clinical trials [23]. Meanwhile, SP activity is regulated by the membrane-bound neutral endopeptidase CD10 (neprilysin), expressed by mesenchymal stem/stromal cells (MSCs) [24,25]. Our previous work demonstrated that IFP-MSCs acquire an immunomodulatory phenotype and degrade SP via CD10 in vitro and in vivo by cleaving SP at the amino side of its hydrophobic residues [26,27]. Notably, CD10High extracellular vesicles (EVs) exhibit miRNA-driven immunomodulatory and anabolic properties that support joint homeostasis [27]. In parallel, we have successfully transduced IFP-MSCs with the CGRP antagonist, CGRP_8-37_, and confirmed that isolated EVs contain the CGRP antagonist (aCGRP IFP-MSC EVs). Importantly, aCGRP IFP-MSC EVs were enriched in miRNAs and proteins known to exert potent anti-inflammatory, immunomodulatory, and cartilage homeostasis effects [28]. These EVs also were able to polarize macrophages away from the pro-inflammatory M1 phenotype towards the anti-inflammatory M2 phenotype.

We hypothesize that aCGRP IFP-MSC EVs attenuate inflammation and pain signaling in vitro and mitigate OA progression in an in vivo rat model of acute synovial/IFP inflammation. Herein, our data support the proposal that aCGRP IFP-MSC EVs can attenuate neuroinflammation of dorsal root ganglia (DRGs) and alter the gene expression of chondrocytes exposed to a pro-inflammatory environment in favor of cartilage anabolism. Also, aCGRP IFP-MSC EV intra-articular infusion results in reduced pain signaling and a preserved cartilage structure.

## 2. Materials and Methods

### 2.1. Isolation, Culture, and Expansion of IFP-MSCs

**Figure 1:**
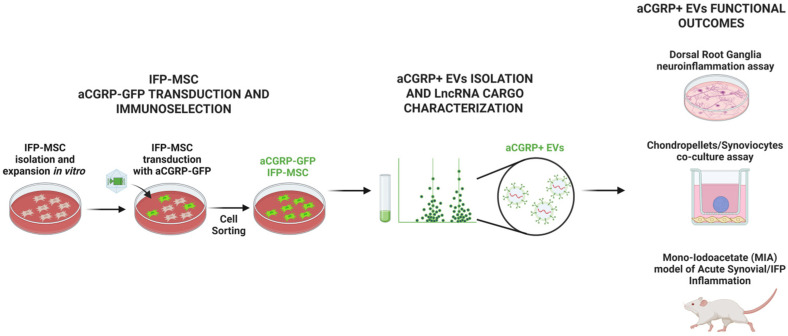
MSCs were isolated from the human infrapatellar fat pad (IFP), transduced with gene construct, and sorted by FACS cell sorting to generate aCGRP IFP-MSCs. aCGRP IFP-MSC EVs were characterized for their LncRNA cargo. EVs were then functionally assessed by a dorsal root ganglia (DRG) neuroinflammation assay, chondropellets/synoviocytes co-culture assay, and acute synovial/IFP inflammation animal model.

All experiments using human cells were performed in accordance with relevant guidelines and regulations. Human IFP-MSCs were isolated from IFP tissue obtained from deidentified, non-arthritic patients (n = 5 donors; two males 26 and 48 years old, and three females 22, 42, and 44 years old) undergoing elective knee arthroscopy at the Lennar Foundation Medical Center at the University of Miami. All procedures were carried out following approval by the University of Miami IRB not as human research (based on the nature of the samples as discarded tissue). The isolation and culture expansion of IFP-MSCs are described in [27] and Figure 1.

### 2.2. Generation of AAV Vector Containing GFP-Labeled CGRP Antagonist Gene

CGRP_8-37_ is a truncated polypeptide of CGRP that serves as a competitive antagonist of CGRP (aCGRP). The generation of an AAV vector containing the GFP-labeled CGRP antagonist gene is described in [27].

### 2.3. IFP-MSC Transduction and Cell Sorting for GFP-Positive aCGRP IFP-MSC

AAV- aCGRP-GFP was used to transduce 20 × 10^6^ P1 IFP-MSC (n = 5 donors). Transduced cultures were maintained at 37 °C, 5% (*v*/*v*) CO_2_ for 7 days, and GFP fluorescence was visualized using a x10 objective Leica DMi8 microscope with Leica X software (Leica, Wetzlar, Germany). On day 7, the transduced IFP-MSCs were sorted based on GFP expression to yield the aCGRP IFP-MSC subpopulation using the MoFlo Astrios EQ cell sorter (Beckman Coulter, Brea, CA, USA) [27]. After sorting, aCGRP IFP-MSCs were seeded on 0.1% gelatin plates (MilliporeSigma, St. Louis, MO, USA) and cultured at 37 °C, 5% (*v*/*v*) CO_2_ until 80% confluency.

### 2.4. Isolating EVs from aCGRP IFP-MSCs

EVs were isolated from aCGRP IFP-MSC conditioned media by a stepwise ultracentrifugation method. The isolation and biophysical and biochemical characterization using flow cytometry and nanoparticle tracking analysis of EVs from aCGRP IFP-MSCs are described in [27]. The IFP-MSC EV yield was 3–4 × 10^9^/mL as detected by nanoparticle tracking analysis for all IFP-MSC donors.

### 2.5. Long Non-Coding RNA (LncRNA) Assay

RNA was isolated from aCGRP IFP-MSC EVs using the Total Exosome RNA and Protein Isolation Kit (Thermo Fisher Scientific, Waltham, MA, USA) according to the manufacturer’s instructions. Then, cDNA was synthesized with an All-in-One miRNA First-Strand cDNA Synthesis Kit (GeneCopoeia, Rockville, MD, USA).

A pre-designed RT2 lncRNA PCR Array Human IncFinder (Qiagen) search was performed using 500 ng cDNA per IFP-MSC sample as per the manufacturer’s instructions and processed using a StepOne Real-time thermocycler (Applied Biosystems, LLC, Waltham, MA, USA). Data analysis was performed using qPCR results with GeneCopoeia’s online Data Analysis System (http://www.genecopoeia.com (accessed on 2 June 2025)). Mean values were normalized to small nucleolar RNA SNORA73 and expression levels were calculated using the 2^−ΔCt^ method. Putative interactomes were generated using a miRNet centric network visual analytics platform (https://www.mirnet.ca/ (accessed on 2 June 2025)). The lncRNA target gene data were collected from a well-annotated database. Values (with 34-cycle cut-off point) were represented in a topology lncRNA–gene interactome network using the force atlas layout and hypergeometric test algorithm.

### 2.6. Dorsal Root Ganglia (DRG) Neuroinflammation Assay

Rat were perfused with 4% paraformaldehyde in 0.1 M PBS, and DRGs were removed and isolated. We mixed 1.0 × 10^5^ TIC inflammatory/fibrotic cocktail (15 ng/mL TNFα, 10 ng/mL IFNγ, 10 ng/mL CTGF)-stimulated DRGs with aCGRP IFP-MSC EVs (at a concentration corresponding to EVs secreted from 1 × 10^6^ IFP-MSC or 3–4 × 10^9^ EVs/mL, n = 2 donors) in each well of a 12-well plate and cultured those for 3 days.

RNA extraction and cDNA synthesis from TIC-stimulated DRGs were performed using the RNeasy Mini Kit (Qiagen) and SuperScript™ VILO™ cDNA synthesis kit (Invitrogen, Waltham, MA, USA), respectively. A predesigned 84-gene rat neuropathic and inflammatory qPCR array (RT2 Profiler neuropathic & inflammatory array, Qiagen) was performed using a StepOne Real-time thermocycler (Applied Biosystems, LLC). Mean values were normalized to ACTB. Expression levels were calculated as the relative fold change in the TIC-stimulated DRGs treated with aCGRP IFP-MSC EVs to TIC-stimulated DRGs alone (reference sample, 2^−ΔCt^ = X sample/X reference sample).

### 2.7. Chondropellets/Synoviocytes Co-Culture Assay

Chondrogenesis was performed by inducing with a serum-free chondrogenic differentiation medium (MesenCult^TM^-ACF Chondrogenic Differentiation Kit, STEMCELL Technologies, Vancouver, BC, Canada) 0.25 × 10^6^ IFP-MSC per pellet for 15 days. After differentiation, the resulting chondropellets were collected and placed in a transwell co-culture system alongside synoviocytes, either with or without the addition of aCGRP IFP MSC EVs (at a concentration corresponding to EVs secreted from 1 × 10^6^ IFP-MSC or 3–4 × 10^9^ EVs/mL, n = 2 donors). The co-cultures were maintained in synoviocyte growth medium supplemented with a pro-inflammatory and fibrotic cytokine cocktail consisting of TNFα (15 ng/mL), IFNγ (10 ng/mL), and CTGF (10 ng/mL) for a duration of 72 h.

After three days, the chondropellets were harvested for histological evaluation and gene expression analysis. For histology, the pellets were cryosectioned at 6 μm thickness and stained using hematoxylin and eosin (H&E) for the general tissue structure and 1% toluidine blue for assessing chondrogenic matrix production. For molecular profiling, RNA was extracted using the RNeasy Mini Kit (Qiagen, Frederick, MD, USA), followed by cDNA synthesis using the SuperScript™ VILO™ kit (Invitrogen). A predesigned 88-gene human osteoarthritis and cartilage repair qPCR array (GeneQuery™ Human Osteoarthritis and Cartilage Repair qPCR Array Kit, ScienCell, Carlsbad, CA, USA) was performed using a StepOne Real-time thermocycler (Applied Biosystems). Mean values were normalized to ACTB. Results were presented as stacked bar graphs, showing fold changes in expression in aCGRP IFP MSC EV-treated pellets relative to untreated controls.

### 2.8. Mono-Iodoacetate (MIA) Model of Acute Synovial/IFP Inflammation

All animal procedures were approved by the Institutional Animal Care and Use Committee (IACUC) at the University of Miami (Approval #21-030 LF) and followed ARRIVE guidelines. A GraphPad Random Numbers calculator was used to randomly assign animals for surgeries and for the subsequent cell treatments within the groups. The order of surgeries was randomized until the appropriate number of animals in each group was reached. Behavioral testing was conducted as stated by an experimenter blinded to the surgical/treatment group. Sigma Stat software v4.0 was used to calculate the sample size for each proposed aim. Input data were used based on our preliminary data with OA and MIA pain models and our experiences with behavioral tests in other pain-related animal models.

Twenty-four male Sprague–Dawley rats were housed individually in a controlled environment (12/12 h light/dark cycle, ad libitum food/water) for one week before being separated into three experimental groups, eight receiving only the MIA injection (negative control), eight receiving CD10High EVs after the MIA injection (positive control), and eight receiving aCGRP EVs after the MIA injection (Supplementary Materials).

Synovial and IFP inflammation was induced via intra-articular injection of 1 mg monoiodoacetate (MIA) in 50 µL saline into the right knee. Under isoflurane anesthesia, injections were administered at 90° knee flexion using the patellar ligament and joint line as anatomical landmarks. Four days later, we performed a single 100 µL intra-articular injection of aCGRP EVs at a concentration corresponding to EVs secreted from 1 × 10^6^ IFP-MSC or 3–4 × 10^9^ EVs/mL.

### 2.9. Behavioral Assessments

**Figure 2:**
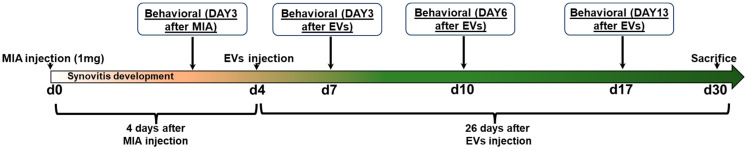
Acute synovial/IFP inflammation often seen in OA was generated by intra-articular injection of MIA into the right knee. Four days later, a single intra-articular injection of aCGRP EVs was performed. Behavioral analysis was performed at baseline, 3 days after MIA injection, and 3, 6, and 13 days post-EV treatment.

Animal well-being was monitored daily post-injections. All behavioral tests were conducted by blinded evaluators. To assess tactile and cold allodynia, rats were placed on a metal mesh platform enclosed in a plastic dome (13 × 17 × 28 cm) and allowed to acclimate for at least 20 min before testing. Behavioral responses were evaluated at baseline, 3 days after MIA injection, and 3, 6, and 13 days post-EV treatment, and then we sacrificed at 30 days after MIA injection (Figure 2).

### 2.10. Tactile Allodynia

Mechanical sensitivity thresholds were determined using von Frey filaments and the up–down method [29]. Filaments were applied to the plantar hind paw, bending slightly upon contact. A withdrawal response, often accompanied by paw licking or body movement, was recorded as positive. Eight calibrated von Frey filaments were used, ranging from 0.25 g to 15 g. A paw withdrawal led to testing with a lower-force filament, while no response prompted testing with a higher-force filament. If the strongest filament (15 g) failed to elicit a response, the threshold was recorded as 15 g. Both hind paws were tested with a 5 min interval between trials.

### 2.11. Cold Allodynia

Cold sensitivity was assessed by counting foot withdrawal responses following the application of an acetone drop to the plantar surface. While uninjured rats typically showed no reaction, injured rats exhibited nociceptive behaviors such as paw shaking or biting. Each paw was tested five times, with a 3–5 min interval between trials. The response frequency was calculated as a percentage.

### 2.12. Knee Bend Test

A modified version of the ankle bend nociception test for monoarthritic rats was used to evaluate knee joint sensitivity during normal movement. Rats were gently restrained to minimize movement while ensuring access to both hind limbs. The experimenter manually flexed and extended each knee joint within its natural range of motion while recording the number of vocalizations (squeaks) and struggle responses. Reactions were scored as follows: 0 for no response, 0.5 for struggle only at maximal flexion/extension, 1 for struggle at moderate flexion/extension or vocalization at maximal flexion/extension, and 2 for squeaking in response to moderate flexion/extension. Each test consisted of five flexions and five extensions, and the total knee bend score (maximum of 20) was used to assess nociceptive sensitivity.

### 2.13. Cytochemical Staining

Knee joints were harvested by excising the femur and tibia/fibula 1 cm above and below the joint line, dissecting soft tissues, and fixing specimens in 10% neutral-buffered formalin (Sigma-Aldrich, St. Louis, MO, USA) for 14 days. Joints were decalcified, sagittally sectioned, embedded in paraffin, and cut into 4 μm serial sections.

Masson’s trichrome staining evaluated the collagen composition. Microscopic imaging (Leica DMi8, x10 objective, Leica X software) was performed on three knees per condition across four fields per knee. Quantitative analysis was conducted using Fiji/ImageJ (v2.14.0).

### 2.14. PRG4 (Lubricin) Immunolocalization

For PRG4 immunofluorescence, sections were permeabilized with PBS + 0.2% Triton X100 (20 min), blocked (PBS + 0.1% Triton X-100 + 1% BSA, 1 h), and incubated overnight at 4 °C with mouse anti-rat PRG4 monoclonal antibody (Sigma, 1:500). After washing (PBS + 0.01% Triton X-100), sections were incubated with Alexa Fluor647-conjugated goat anti-mouse IgG (Thermo Fisher) for 2 h. Controls received secondary antibody only. Sections were counterstained with DAPI (Invitrogen). Microscopic imaging (Leica DMi8, x10 objective, Leica X software) was performed on three knees per condition across four fields per knee. Quantitative analysis was conducted using Fiji/ImageJ (v2.14.0).

### 2.15. Substance P Immunolocalization

For anti-substance P immunofluorescence staining, tissue sections were first incubated in 1× citrate buffer at 60 °C overnight for antigen retrieval. The following day, sections were permeabilized with 1× PBS containing 0.2% Triton X-100 for 20 min at room temperature, then blocked in a buffer composed of 1× PBS, 0.1% Triton X-100, and 10% rabbit serum for 1 h at room temperature. Between each step, sections were washed with 1× PBS. Sections were then incubated overnight at 4 °C with rabbit anti-rat substance P polyclonal antibody diluted 1:100 in blocking buffer. After washing with 1× PBS containing 0.01% Triton X-100, sections were incubated for 1 h at room temperature with an Alexa Fluor 594-conjugated goat anti-rabbit IgG secondary antibody (Thermo Fisher Scientific). Control sections were treated with the secondary antibody only. Finally, all sections were rinsed with 1× PBS and mounted using ProLong Gold Antifade Reagent with DAPI (Invitrogen). Microscopic imaging (Leica DMi8, x10 objective, Leica X software) was performed on three knees per condition across four fields per knee. Quantitative analysis was conducted using Fiji/ImageJ (v2.14.0).

### 2.16. Statistical Analysis

The Kolmogorov–Smirnov normality test was used to assess whether there was a normal distribution of values. For multiple comparisons, one-way or two-way ANOVA was used in GraphPad Prism v7.03 (GraphPad Software, San Diego, CA, USA) with statistical significance at *p* < 0.05.

## 3. Results and Discussion

### 3.1. aCGRP IFP-MSC EV LncRNA Cargo

Long non-coding RNAs (lncRNAs) are emerging as key epigenetic regulators in skeletal system remodeling, particularly through their influence on cell MSC differentiation. By modulating the balance between osteogenic and adipogenic pathways, lncRNAs play a central role in bone homeostasis, regeneration, and cartilage integrity. Recent studies highlight the complex interplay between lncRNAs and miRNAs, which together coordinate gene expression programs involved in osteoarthritis pathogenesis.

The gene construct of an adenovirus vector to transduce IFP-MSCs, demonstration of parental aCGRP+ IFP-MSC cell sorting efficiency, their clonogenic capacity (stemness), and immunophenotypic profiling are reported in [27]. For aCGRP IFP-MSC EVs, the biophysical and biochemical characterization using flow cytometry and nanoparticle tracking analysis are described in [27].

**Figure 3:**
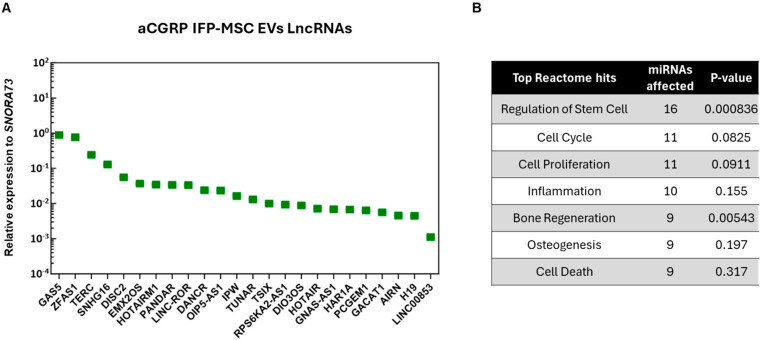
(**A**) Twenty-four distinct LncRNAs were present as cargo in aCGRP IFP-MSC EVs. Mean values were normalized to small nucleolar RNA SNORA73. (**B**) LncRNAs present in aCGRP IFP-MSC EVs were involved in the regulation of important cell processes, such as regulation of the stem cells, cell cycle, cell proliferation, inflammation, bone regeneration, osteogenesis, and cell death. Putative interactomes were generated using a miRNet centric network visual analytics platform.

In the present study, out of the 96 LncRNAs investigated, we detected 24 unique LncRNAs in the aCGRP EVs. Their levels of relative expression ranged from 0.88 for GAS5 to 0.001106 for LINC000853 (Figure 3). Pathway analysis revealed that most detected LncRNAs are involved in important cell processes such as regulation of the stem cells, cell cycle, cell proliferation, inflammation, bone regeneration, osteogenesis, and cell death. However, seven LncRNAs showed no specific regulatory effects on known miRNAs related to the OA pathogenesis.

Specifically, 11 LncRNAs (ZFAS1, EMX2OS, HOTAIRM1, RPS6KA2-AS1, DANCR, LINC-ROR, GACAT1, GNAS-AS1, HAR1A, OIP5-AS1, TERC) interact with miRNAs that promote cell proliferation, prevent apoptosis, and preserve homeostasis. Firstly, ZFAS1 LncRNA has been shown to protect chondrocytes from apoptosis and extracellular matrix degradation by sponging miR-7-5p to regulate FLRT2 expression [30]. EMX2OS LncRNA has been known to interact with miR-653-5p and decrease its effects on cartilage homeostasis. Specifically, miR-653-5p is known for playing a significant role in OA by modulating chondrocyte senescence [31]. In another study, the downregulation of HOTAIRM1 LncRNA inhibited MSC viability, induced apoptosis, and suppressed differentiation. This could be the result of HOTAIRM1 regulating the miR-125b/BMPR2 axis [32]. RPS6KA2-AS1 LncRNA is associated with gene RPS6KA2, which is involved in chondrocyte differentiation and cartilage formation. Increased RSPS6KA could promote MSC proliferation and chondrogenic differentiation [33]. Importantly, DANCR LncRNA with miR-320a can induce proliferation and chondrogenesis by regulating the Wnt/β-catenin signaling pathway and inhibiting CTNNB1 gene expression during osteogenic differentiation [34]. LINC-ROR LncRNA has been shown to interact with miR-138 and miR-145, and as a result, induce SOX9 gene expression, MSC chondrogenesis, and cartilage formation [35]. The inhibition of GACAT1 LncRNA has been shown to inhibit the proliferation and migration of oral squamous carcinoma cells, suggesting that GACAT1 plays a role in cell proliferation [36]. Similarly, when GNAS-AS1 LncRNA was inhibited in an osteosarcoma study, cell proliferation, migration, and invasion were also inhibited [37]. Next, HAR1A LncRNA has been shown to have immunomodulatory effects by binding to miR-149-3p and suppressing its expression [37]. OIP5-AS1 LncRNA interacts with miR-29b-3p, which promotes chondrocyte proliferation and migration and inhibits apoptosis and inflammation [38]. And lastly, TERC LncRNA has a role in bone metabolism by absorbing miRNA-217, which in turn upregulates RUNX2 gene expression [39].

In addition, six LncRNAs (GAS5, TSIX, PCGEM, H19, HOTAIR, SNHG16) have been linked to miRNA regulation, which prevents cell proliferation, promotes apoptosis, and dysregulates cell homeostasis. A study found that GAS5 LncRNA is upregulated in OA patients, as a result inducing chondrocyte apoptosis and inhibiting chondrocyte proliferation by downregulating miR-137 [40]. Also, TSIX LncRNA can be upregulated in OA patients, suggesting a role in chondrocyte dysfunction by interacting with miR-320a. Inhibiting TSIX LncRNA leads to enhanced cell viability and mitigated IL-1B-induced apoptosis [41]. A study found that PCGEM LncRNA is upregulated in synoviocytes of OA patients. Specifically, PCGEM LncRNA interacts with miR-142-5p, resulting in the upregulation of RUNX2 gene expression [42]. Next, H19 LncRNA was shown to interact with miR-130a. H19 LncRNA knock-out resulted in increased viability, decreased apoptosis, and reduced inflammatory factor secretion [43]. HOTAIR LncRNA was shown to have an antagonistic relationship with miR-17-5p, and upon HOTAIR upregulation, miR-17-5p expression is downregulated, leading to increased apoptosis [44]. Lastly, SNHG16 LncRNA was shown to interact with miR-373-3p. SNHG16 LncRNA knock-out results in an increase in miR-373-3p, decreasing IL-1B-induced apoptosis, which implies that SNHG16 regulates miR-373-3p [45].

### 3.2. aCGRP IFP-MSC EVs’ Effects on Dorsal Root Ganglia Under Inflammatory Conditions

**Figure 4:**
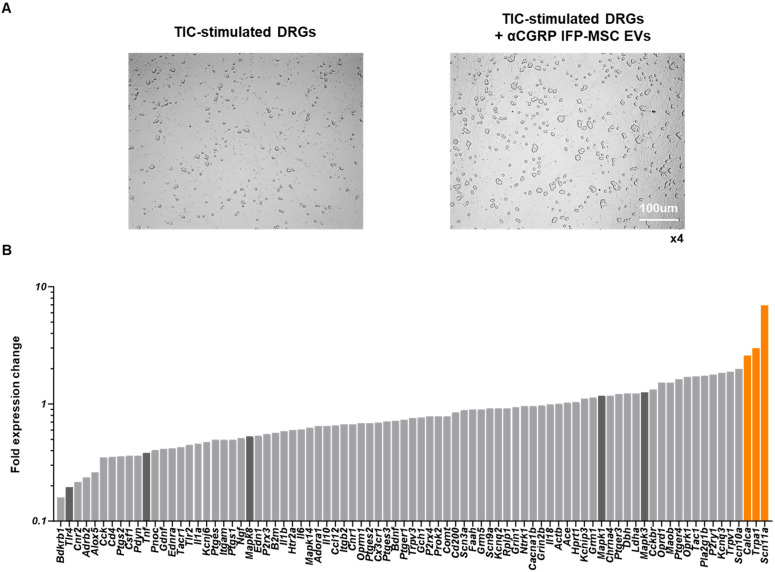
Dorsal root ganglia (DRG) neuroinflammation assay. (**A**) DRGs showed identical morphology with and without the addition of aCGRP IFP-MSC EV treatment. (**B**) TIC-stimulated DRG molecular profiling indicated an overall reduced neuroinflammatory profile upon exposure to aCGRP IFP-MSC EVs. Out of the 84 genes, only 3 (Calca, Trpa1, and Scn11, highlighted orange) showed increased expression (>2 fold) compared to TIC-stimulated DRGs alone. Five important genes (Mapk1, Mapk3, Mapk8, Tlr4, and Tnf, highlighted dark gray) involved in neuroinflammation were highly downregulated (>2-fold) upon exposure to aCGRP IFP-MSC EVs. White scale bar: 100 μm. Magnification ×4.

Our preliminary studies have shown that CGRP_8-37_ (truncated CGRP peptide) acts as a CGRP antagonist, temporarily reversing migraine and neuropathic pain symptoms in animal models [16,17,18,19,20,21,22]. Specifically, AAV_CGRP_8-37_ intraspinal or intrathecal injection can decrease chronic neuropathic pain in rat spinal cord injury and peripheral nerve injury pain models. Herein, DRGs showed an identical morphology with and without the addition of aCGRP IFP-MSC EV treatment (Figure 4A). TIC-stimulated DRGs’ molecular profiling indicated an overall reduced neuroinflammatory profile upon exposure to aCGRP IFP-MSC EVs (Figure 4B). Out of the 84 genes investigated, only 3 (Calca, Trpa1, and Scn11a) showed increased expression (>2 fold) compared to TIC-stimulated DRGs alone. Interestingly, Calca, which is the gene for CGRP, is a classic marker of nociceptive DRGs. The high Calca gene expression levels could be the result of aCGRP being recognized as CGRP by the qPCR assay, but more research is needed to prove this hypothesis [46]. The transient receptor potential ankyrin 1 (TRPA1) has been shown to be involved in inflammatory responses, and in the conversion of physical and chemical stimuli in irritative or pain sensations [47]. Lastly, Scn11a encodes the tetrodotoxin-resistant (TTX-R) sodium channel Nav1.9a/NaN, which is preferentially expressed in nociceptive primary sensory neurons of DRGs [48].

Five important genes (Mapk1, Mapk3, Mapk8, Tlr4, and Tnf) involved in neuroinflammation were highly downregulated (>2-fold) upon exposure to aCGRP IFPMSC EVs. In this context, mitogen-activated protein kinases (MAPKs) are involved in inflammation [49,50] and modulate nociceptive signaling and peripheral/central sensitization [51], whereas their inhibition results in anti-inflammatory effects in inflammatory diseases [49,52]. Different MAPK isoforms, such as MAPK1 and MAPK8, contribute to pain sensitization via both neuronal and glial mechanisms. These two isoforms were downregulated following EV treatment (Figure 4B). MAPK8 activation in astrocytes contributes to the maintenance of chronic pain [53]. MAPK1 alone can induce inflammatory pain, whereas with MAPK3, it results in sensory neuron survival [54]. Also, TLR4 is a pattern recognition receptor, and its activation on spinal microglia and astrocytes leads to the production of pro-inflammatory cytokines, including TNF, and contributes to the development of pain. TLR4 activation has been shown to induce tactile allodynia through the release of prostaglandin E2 (PGE2) and TNF, which are critical mediators of pain signaling [55]. Additionally, TLR4 activation enhances calcium signaling in astrocytes, promoting cytokine production and further promotion of neuroinflammation and pain [56]. Finally, TNF is a pro-inflammatory cytokine that plays a pivotal role in the pathogenesis of neuroinflammation and pain. TNF is produced by activated glial cells in response to TLR4 activation and contributes to the sensitization of nociceptive pathways [57]. TNF-dependent pathways are involved in the maintenance of persistent pain states, highlighting its importance in chronic pain conditions.

### 3.3. Effects of aCGRP IFP-MSC EVs on Chondrocytes Under Inflammatory Conditions

**Figure 5:**
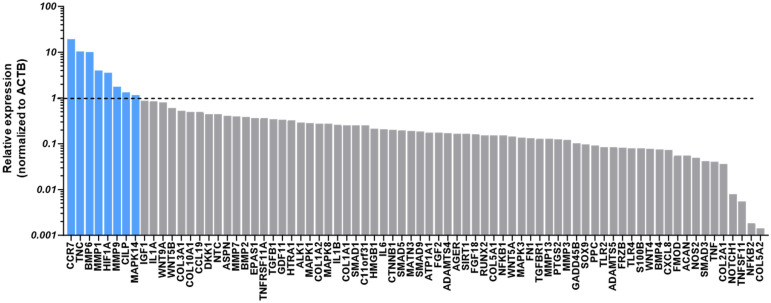
Chondropellets/synoviocytes co-cultures under inflammatory conditions. Treatment of chondropellets/synoviocytes co-cultures with aCGRP IFP-MSC EVs led to upregulated expression of several genes in chondropellets with diverse roles in cartilage biology, some of which are closely tied to anabolic processes, while others are typically associated with catabolic or inflammatory functions. Dotted line marks the gene expression levels without EV treatment (TIC-stimulated chondropellets/synoviocytes alone). Gray bars: gene expression below the threshold, blue bars: gene expression above threshold.

Treatment of chondropellets/synoviocytes inflammatory co-cultures with aCGRP IFP-MSC EVs led to upregulated expression of several genes in chondropellets with diverse roles in cartilage biology, some of which are closely tied to anabolic processes, while others are typically associated with catabolic or inflammatory functions (Figure 5). Notably, the upregulation of BMP6 and HIF1A gene expression suggests a pro-anabolic effect of the EV treatment. BMP6, a member of the TGF-β superfamily, is well recognized for its ability to stimulate chondrogenesis, promote extracellular matrix (ECM) synthesis, and enhance the expression of key anabolic markers such as aggrecan and collagen type II [58]. Similarly, HIF1A is a master regulator of cellular adaptation to hypoxia, a condition inherent to the avascular cartilage environment. HIF1A supports anabolic activity by promoting chondrocyte survival and upregulating SOX9, a critical transcription factor for cartilage matrix synthesis [59,60,61]. These gene expression changes suggest that aCGRP IFP-MSC EVs may create a microenvironment conducive to cartilage repair.

Interestingly, the EV-induced upregulation of TNC (Tenascin-C) gene expression may also play a supportive role in anabolism, particularly in the early stages of cartilage remodeling. TNC is known to be upregulated during tissue injury and repair and can enhance cell migration and adhesion [62,63]. However, it also has context-dependent effects, as sustained TNC expression may contribute to inflammation through Toll-like receptor activation, potentially limiting its long-term anabolic benefits [63]. Likewise, Cartilage Intermediate Layer Protein (CILP) has been shown to inhibit insulin-like growth factor-1 (IGF-1) signaling, which is crucial for anabolic responses in cartilage [64]. While its expression is part of a normal ECM composition, high levels could dampen IGF-1-mediated repair.

More surprising was the upregulation of MMP1 and MMP9 gene expression, which traditionally is associated with matrix degradation (Figure 5). These metalloproteinases are commonly elevated in OA tissue and contribute to cartilage breakdown by degrading collagens and other ECM components [65]. However, transient or controlled expression of MMPs may be necessary for matrix remodeling and the removal of damaged ECM components prior to repair, particularly during early phases of regeneration [66]. Their induction by EVs might therefore reflect a preparatory remodeling response rather than overt degeneration. Similarly, MAPK14 (p38 MAPK) gene expression exhibits a dual role in chondrocyte biology, where it can support anabolic activity by stabilizing and upregulating SOX9 under certain conditions, such as TGF-β stimulation, but also promote catabolic responses through inflammatory signaling and chondrocyte apoptosis in other contexts [67,68].

Lastly, the observed upregulation of CCR7 gene expression, a chemokine receptor typically associated with immune cell recruitment and inflammation, calls for important considerations. While traditionally viewed as a contributor to synovial inflammation in OA, recent evidence suggests that chemokine signaling can also participate in tissue repair by modulating cell migration and intercellular communication [69,70]. Its role in EV-mediated cartilage regeneration remains to be clarified, but it may reflect the broader immunomodulatory effects of EVs on the joint environment.

Taken together, the EV-induced gene expression profile observed in the current study suggests a coordinated response that may initially involve ECM remodeling and immune modulation, followed by activation of anabolic pathways critical for cartilage repair. While some upregulated genes are associated with catabolic or inflammatory functions in chronic OA, their controlled activation in vitro may reflect a regenerative rather than degenerative program, warranting further investigation into the temporal dynamics and dose-dependency of EV-mediated effects. The therapeutic potential of EVs could also be further optimized through preconditioning/priming in vitro strategies. For example, our studies showed that priming parental MSCs with oxytocin and inflammatory cues significantly enrich EVs’ miRNA cargo, resulting in upregulation of FMOD, TIMP1, CXCL8, SOX9, IGF1, and BMP4 genes’ expression in chondrocytes linked to cartilage matrix remodeling and chondroprotection [71]. These changes are accompanied by enhanced activation of pathways related to connective tissue development and extracellular matrix organization [71].

### 3.4. aCGRP IFP-MSC EV Effects on Pain Sensitization Assessed by Behavioral Testing

**Figure 6:**
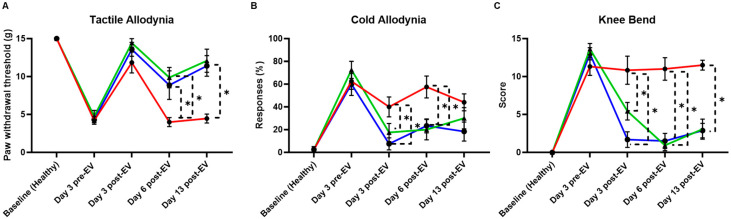
Behavioral assessment of MIA model of acute synovial/IFP inflammation. (**A**) The tactile allodynia (von Frey filament tactile test) evaluated the paw withdrawal threshold. aCGRP IFP-MSC EV-treated knees showed lower allodynia compared to non-treated. (**B**) The cold allodynia (acetone test) showed the %response of EV-treated and non-treated knees to acetone exposure. aCGRP IFP-MSC EV-treated knees showed lower %response compared to non-treated. (**C**) The knee bend test evaluated the pain score upon knee bend. aCGRP IFP-MSC EV-treated knees showed lower pain score compared to non-treated. Blue lines: aCGRP IFP-MSC EV-treated animals, red lines: diseased animals (negative control), green lines: CD10High IFP-MSC EV-treated animals (positive control). Individual black points represent mean ± SEM, * *p* < 0.05.

The behavioral outcomes observed following MIA injection support the development of an inflammatory arthritis phenotype characterized by joint inflammation, cartilage degradation, and persistent pain. MIA induces chondrocyte death and disrupts cartilage metabolism, mimicking the degenerative processes seen in human OA. In this model, non-treated animals exhibited heightened pain sensitivity across multiple modalities (Figure 6). The acetone test revealed increased cold allodynia, the knee bend test demonstrated enhanced joint-related pain responses, and the cold hypersensitivity assay showed decreased paw withdrawal thresholds (marked with red lines). All behavioral tests reflect the development of central and peripheral sensitization that typically accompanies chronic joint degeneration.

Remarkably, animals treated with EVs displayed attenuated pain behaviors across all tests. aCGRP IFP-MSC EV-treated knees showed a significantly reduced responsive-ness to acetone application, indicating a decrease in cold sensitivity. Significant differences in the pain withdrawal threshold existed in von Frey filaments tested six days after EV treatment and persisted at the last timepoint after EV treatment. Meanwhile, the EV-treated groups had significantly reduced knee bend scores, starting three days after EV treatment and lasting until the final timepoint. Lower pain scores in the knee bend test further suggest reduced mechanical joint pain, and the improved withdrawal thresholds in the von Frey tactile test highlight a broader analgesic effect. These findings suggest that EV therapy may counteract key pathophysiological features of OA, such as neuroinflammation and aberrant nociceptive signaling. Given that pain is one of the most debilitating features of OA, the ability of EVs to modulate these behavioral responses underscores their potential as a disease-modifying therapeutic, capable not only of addressing inflammation and tissue repair but also mitigating the chronic pain associated with joint injury.

### 3.5. aCGRP IFP-MSC EV Effects on Cartilage Homeostasis and Pain Signaling Assessed by Histological Evaluation

**Figure 7:**
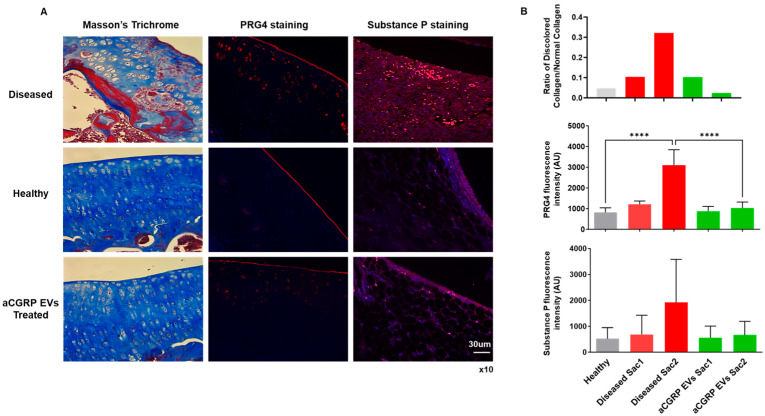
Histochemical and immunohistochemical evaluation of chondroprotective and analgesic effects of aCGRP IFP-MSC EVs’ intra-articular administration in rat knee joint. (**A**) Masson’s trichrome staining revealed pronounced collagen disruption (red staining) in diseased cartilage (top panel) compared to healthy (middle panel) and treatment (bottom panel) groups. Immunohistochemical staining for PRG4 illustrated upregulation of PRG4 expression to the intermediate zone in MIA-only group compared to treatment and healthy groups. Immunohistochemical staining for SP illustrated greater signal (red) in diseased animals compared to treatment and healthy groups. White scale bar: 30 μm. Magnification ×10. (**B**) Treatment with aCGRP IFP-MSC EVs significantly improved collagen preservation, with a lower ratio of discolored to normal collagen. Treatment with aCGRP IFP-MSC EVs significantly lowered quantity of PRG4 expressed at the second sacrifice. Immunofluorescence analysis showed elevated SP levels in diseased cartilage compared to healthy controls. Although EV-treated samples showed a non-significant reduction in SP, these trends suggest potential modulation of neurogenic inflammation. Gray bars: healthy knees, red bars: diseased knees sacrifice 1 (Sac1) and sacrifice 2 (Sac2), green bars: EV-treated knees sacrifice 1 (Sac1) and sacrifice 2 (Sac2). **** *p* < 0.0001.

Histochemical and immunohistochemical evaluation revealed chondroprotective and analgesic effects upon aCGRP IFP-MSC EVs’ intra-articular administration in rat knee joints (Figure 7). Masson’s trichrome staining has been effectively employed as a histochemical technique to assess alterations in collagen composition [72]. In this study, cartilage from the disease group exhibited pronounced red staining, indicating collagen disruption, when compared to other experimental groups. Notably, it appears to be a progression of the disease in the untreated group from the timepoint of the first sacrifice to the timepoint of the second as the ratio of discolored to normal cartilage increases. Remarkably, administration of aCGRP IFPMSC EVs led to a significant enhancement in the normal collagen content, with a lower ratio of discolored to normal collagen, closely resembling staining of the healthy control group (Figure 7A,B).

To explore the phenotypic impact of aCGRP IFP-MSC EV administration, we conducted immunohistochemical analysis of PRG4 expression in situ (Figure 7A,B). In healthy cartilage, PRG4+ cartilage-derived stem/progenitor cells (CSPCs) formed a well-defined band in the superficial layer. In contrast, diseased cartilage exhibited some PRG4 expression in this zone, coupled with a significant upregulation in the intermediate zone, where chondrocytes likely increase PRG4 production to compensate for superficial layer loss during disease progression. Importantly, the expression of PRG4 in the aCGRP IFP-MSC EV-treated group was comparable to that of the healthy group, with no significant difference between the healthy and treated groups at the second timepoint of sacrifice.

Finally, to analyze the effects of aCGRP IFP-MSC EV administration on SP degradation, we performed an immunofluorescence test on synovium/IFP tissues (Figure 7A,B). Using healthy tissue as a baseline, we compared the SP presence of the diseased samples and the EV-treated samples at different end points in the experiment. Even though not statistically significant, the aCGRP IFP-MSC EV-treated group showed a decreased SP presence in synovium/IFP tissues. Moreover, SP not only plays a key role in nociception but also modulates local neurogenic inflammation and immune responses. It increases vascular permeability to promote immune cell infiltration and influences macrophage migration and polarization at sites of inflammation [4,6,7,8]. Given that aCGRP IFP-MSC EVs are derived from parental MSCs that highly express CD10 (neprilysin), an endopeptidase that cleaves Substance P [25,27,73,74], we expect a decreased SP presence in the joint capsule.

These findings provide compelling evidence for the therapeutic potential of aCGRP IFPMSC EVs in the context of OA. OA is characterized by cartilage matrix disruption, chronic inflammation, and failed repair responses due to the limited regenerative capacity of the joint. The observed restoration of the collagen content and structure following EV treatment, as shown by Masson’s trichrome staining, suggests a protective or reparative effect on extracellular matrix integrity, a critical component often compromised in OA. Furthermore, the preservation and relocalization of PRG4+ CSPCs in the superficial zone imply that EVs may help maintain the endogenous repair machinery disrupted in disease. Also, the decreased SP presence in the EV-treated group, along with the known role of SP in promoting neurogenic inflammation and macrophage recruitment, supports the hypothesis that neprilysin-expressing EVs could attenuate pain cascades associated with OA. Together, these results highlight a multifaceted mechanism by which aCGRP IFP-MSC EVs may mitigate joint degeneration.

## 4. Conclusions

Clinically, nonoperative treatment for osteoarthritis (OA) typically progresses from lifestyle modification to oral and topical NSAIDs, to intra-articular injections. NSAIDs remain a first-line therapy due to their consistent efficacy in reducing pain and improving function, with both oral and topical forms strongly supported by AAOS and ACR guidelines [75]. However, their long-term use is limited by gastrointestinal, renal, and cardiovascular risks [76,77]. When oral medications fail, intra-articular corticosteroid injections are often the next step, providing meaningful but short-term relief—typically lasting a few weeks to months. They are effective for inflammatory flares, yet concerns remain about potential cartilage damage with frequent use and systemic side effects such as transient hyperglycemia [76,77,78]. Hyaluronic acid injections aim to restore joint lubrication and viscoelasticity but show inconsistent efficacy across studies, and both AAOS and ACR generally discourage their routine use due to limited and variable evidence [75]. Platelet-rich plasma (PRP) therapy has emerged as a promising biologic option, with several randomized trials and meta-analyses suggesting superior symptom improvement compared with saline or hyaluronic acid, particularly in earlier stages of OA. Still, the lack of standardization in PRP preparation, inconsistent results, and limited insurance coverage temper its widespread adoption, leading AAOS to issue only a limited recommendation [75]. MSC and bone-marrow aspirate concentrate (BMAC) injections represent another frontier, targeting biologic repair and anti-inflammatory modulation, yet evidence remains low-quality and heterogeneous, with uncertain long-term safety and high cost—thus, major societies recommend use only within clinical trials [79,80]. Across all injection therapies, the consistent limitation is durability—most provide transient symptom relief without altering disease progression. Emerging work on EV-based therapeutics suggests the potential to deliver sustained anti-inflammatory and immunomodulatory effects, offering a more durable, disease-modifying approach [81].

The promise of EVs in OA is tempered by some limitations in terminology, methodology, and clinical translation. The field is still young, and even basic definitions remain inconsistent; most clinical trials still use the term “exosomes,” despite updated ISEV guidelines recommending “extracellular vesicles” as the preferred umbrella term and discouraging biogenesis-based labels unless the origin is confirmed. This lack of standardized nomenclature parallels broader reporting variability—few trials disclose their EV isolation methods or provide adequate characterization—making it difficult to reproduce findings or compare therapeutic effects across studies [82,83]. Technical challenges further complicate translation: widely used methods like ultracentrifugation are inexpensive but can damage vesicles, while higher-purity approaches such as density gradients or size-exclusion chromatography vary in scalability and yield [84]. Even when EVs are successfully produced, major clinical uncertainties remain, including a heterogeneity of yielded EVs, potency, optimal dosing, route of administration, long-term safety, immunogenicity, and off-target distribution [85]. For OA specifically, retaining EVs within dense, avascular cartilage and achieving meaningful cellular uptake are persistent obstacles, and current dosing regimens vary widely; although recent early-phase trials have begun exploring dose optimization for knee OA, a therapeutic window has yet to be defined [86,87,88,89]. Emerging strategies such as intranasal, intramuscular, and scaffold-based delivery may improve tissue targeting, but their relevance to human OA remains unclear [90]. Finally, a significant translational gap persists: rodent joints differ substantially from human joints in cartilage thickness, loading patterns, and inflammatory milieu, limiting the predictive value of preclinical studies [91]. Collectively, these challenges highlight the need for standardized EV characterization, improved isolation methods, and more representative OA models before EV-based therapies can be reliably integrated into clinical care.

In the present study, we have successfully generated under regulatory-compliant conditions EVs from genetically modified with the CGRP antagonist IFP-MSC. Specifically, we have shown that aCGRP IFP-MSC EVs exert potent immunomodulatory, analgesic, and chondroprotective effects in an animal model of OA. In stimulated DRGs, EVs downregulated key mediators of neuroinflammation and nociceptive sensitization, including MAPK1/8, TLR4, and TNF, indicating their role in dampening chronic pain signaling pathways. Behaviorally, aCGRP IFP-MSC EV-treated animals exhibited marked reductions in pain behaviors, reflecting functional improvements consistent with reduced peripheral and central sensitization. Histologically, aCGRP IFP-MSC EV administration improved cartilage matrix integrity, restored superficial-zone CSPC organization, and showed reduced SP levels. Collectively, these findings support the therapeutic potential of aCGRP IFP-MSC EVs as a multi-modal intervention capable of addressing inflammation, pain, and structural degradation in OA, highlighting their promise as a disease-modifying treatment.

## Data Availability

The original contributions presented in this study are included in the article/Supplementary Materials. Further inquiries can be directed to the corresponding author.

## Supplementary Materials

The following supporting information can be downloaded at: https://www.mdpi.com/article/10.3390/cells14241952/s1.
