# Supplementary material for: Functional Assessment of Genetically Modified Infrapatellar Fat Pad Mesenchymal Stem/Stromal Cell-Derived Extracellular Vesicles (EVs): Potential Implications for Inflammation/Pain Reversal in Osteoarthritis
Source: Cells. 2025 Dec 9;14(24):1952. doi: 10.3390/cells14241952 (PMC12731162; doi:10.3390/cells14241952)
Supplement: Supplementary file 1 [file cells-14-01952-s001.zip › cells-3806185-supplementary.docx]

**Supplementary Materials:**

| Group | # of animal | Injection Received |
| --- | --- | --- |
| Diseased  animals  (negative control) | 1 | No injection |
|  | 2 | No injection |
|  | 3 | No injection |
|  | 4 | No injection |
|  | 5 | No injection |
|  | 6 | No injection |
|  | 7 | No injection |
|  | 8 | No injection |
| CD10High  EVs-treated  animals  (positive control) | 1 | CD10High |
|  | 2 | CD10High |
|  | 3 | CD10High |
|  | 4 | CD10High |
|  | 5 | CD10High |
|  | 6 | CD10High |
|  | 7 | CD10High |
|  | 8 | CD10High |
| aCGRP  EVs-treated  animals | 1 | aCGRP |
|  | 2 | aCGRP |
|  | 3 | aCGRP |
|  | 4 | aCGRP |
|  | 5 | aCGRP |
|  | 6 | aCGRP |
|  | 7 | aCGRP |
|  | 8 | aCGRP |
